# Microstructure Evolution and Mechanical Stability of Retained Austenite in Medium-Mn Steel Deformed at Different Temperatures

**DOI:** 10.3390/ma12183042

**Published:** 2019-09-19

**Authors:** Aleksandra Kozłowska, Aleksandra Janik, Krzysztof Radwański, Adam Grajcar

**Affiliations:** 1 Institute of Engineering Materials and Biomaterials, Silesian University of Technology, 18A Konarskiego Street, 44-100 Gliwice, Poland; aleksandra.kozlowska@polsl.pl; 2 Łukasiewicz Research Network-Institute for Ferrous Metallurgy, 12-14 K. Miarki Street, 44-100 Gliwice, Poland; ajanik@imz.pl (A.J.); kradwanski@imz.pl (K.R.)

**Keywords:** medium manganese steel, bainitic steel, TRIP effect, retained austenite, mechanical stability, deformation temperature

## Abstract

The temperature-dependent microstructure evolution and corresponding mechanical stability of retained austenite in medium-Mn transformation induced plasticity (TRIP) 0.17C-3.1Mn-1.6Al type steel obtained by thermomechanical processing was investigated using scanning electron microscopy (SEM), electron backscatter diffraction (EBSD), and X-ray diffraction (XRD) techniques. Specimens were deformed up to rupture in static tensile tests in the temperature range 20–200 °C. It was found that an increase in deformation temperature resulted in the reduced intensity of TRIP effect due to the higher stability of retained austenite. The kinetics of strain-induced martensitic transformation was affected by the carbon content of retained austenite (RA), its morphology, and localization in the microstructure.

## 1. Introduction

A new concept of lean medium-Mn transformation induced plasticity (TRIP) steels containing 3–5 wt.% manganese is being intensively developed due to growing quality requirements and cost effectiveness of the automotive industry. Medium-Mn steels are characterized by superior mechanical properties, higher than multiphase ferritic–bainitic steels with retained austenite (first generation of Advanced High Strength Steels—AHSS) and at lower production costs compared to the high-Mn austenitic steels (second generation AHSS) [1,2,3]. 

Earlier studies concerning multiphase advanced high-strength steels [4,5,6] revealed that retained austenite was a key microstructural constituent which provided the outstanding strength–ductility balance. The gradual transformation of metastable austenite into martensite triggered by deformation resulted in the significant work hardening and delayed necking. A multiphase microstructure of medium-Mn steels composed of retained austenite located in the ferritic, bainitic, or martensitic matrix can be obtained as cold-rolled or thermomechanically processed sheets [7,8,9].

C and Mn are known as important austenite stabilizers. Therefore, it seems that increasing content of these elements will increase the amount of retained austenite (RA) at room temperature. Unfortunately, an increase in the carbon content caused a deterioration of weldability [10] and may lead to an occurrence of plastic instability phenomenon—Portevin–Le Chatelier (PLC effect) [11,12]. Stabilization of the γ phase obtained by Mn addition also had some disadvantages. Sun et al. [13] reported that increasing the Mn amount from 7% to 10% resulted in the reduction of the mechanical stability of retained austenite due to the lower C enrichment of the retained austenite. In addition to the chemical factors affecting the stability of RA, other factors such as grain size, morphology, stress state, strain rate, and temperature also had an influence on the TRIP effect efficiency [4,14,15,16,17,18].

During industrial operations, the deformation temperature plays an important role affecting mechanical properties of steel products. Min et al. [15] reported that strain rates in automotive stamping processes can reach 1–10 s^−1^ with minimal heat dissipation. Rusinek [14] reported that during stamping the temperature can reach even ~280 °C. However, usually the temperature under normal operating conditions did not exceed ~130 °C [14]. Therefore, chemical composition and microstructure of steel should be designed for providing the maximum TRIP effect at the specific deformation temperatures. For example, in Fe-0.11%C-0.62%Si-1.65%Mn steel, total elongation of ca. 30%–35% was achieved during tensile tests in a temperature range 15–110 °C [17]. Fe-0.12C-10Mn-2Al-0.05Si steel investigated by Zhang et al. [18] showed an optimal TRIP effect in the temperature range 25–50 °C, while increasing the deformation temperature to 100 °C resulted in noticeable deterioration of yield stress (YS), ultimate tensile strength (UTS), and total elongation (TE) values. De Cooman [16] suggested that for automotive applications TRIP steels should be designed with room temperature located between the M_s_ and M_d30_ temperature range, whereas M_d30_ should be below 100 °C.

The present study concerned the microstructure evolution and mechanical stability of retained austenite in a medium-Mn TRIP steel at various deformation temperatures. It required a proper identification of retained austenite, which was quite difficult due to its relatively small size, varying morphology, and its distribution in the matrix. XRD was a commonly used method to estimate the amount of RA in AHSS showing a TRIP effect. However, this method had some limitations. Therefore, the electron backscatter diffraction (EBSD) method was also applied to show a general tendency. EBSD in comparison to the XRD method was not affected by a crystallographic orientation or texture [19,20,21]. Moreover, it can provide not only the amount of retained austenite but also distribution and morphology of the γ phase.

## 2. Material and Experimental Techniques

### 2.1. Material and Processing Parameters

Investigations were performed on 0.17C-3.1Mn-1.6Al-0.22Si-0.22Mo-0.04Nb (wt.%) TRIP steel characterized by high metallurgical purity (0.005 wt.% S, 0.008 wt.% P). A Nb microaddition was included to increase the strength through grain refinement and precipitation strengthening. Mo was added for solid solution strengthening. Relatively low C and Mn contents were beneficial due to lower probability of plastic instability phenomenon (PLC effect) occurrence [11,12].

The medium-Mn steel was melted by means of vacuum induction furnace under Ar atmosphere (Institute for Ferrous Metallurgy, Gliwice, Poland). The ingots were homogenized at 1200 °C for 3 h in order to eliminate segregation of chemical composition. The next step included hot forging in a temperature range between 1200 and 900 °C. Then, the steel was hot rolled in four passes to obtain flat samples 9 mm × 170 mm × 500 mm. Final thermomechanical treatment consisted of three passes and the final deformation temperature was circa 850 °C (sheet thickness ~4.5 mm). Then, the flat steel samples were cooled (10 °C/s) to 700 °C and held at this temperature within 15 s to induce the austenite–ferrite transformation. Next, accelerated cooling (27 °C/s) to the bainitic transformation temperature 400 °C was applied. Isothermal holding time at 400 °C was 300 s. Finally, air cooling to room temperature was applied. The detailed information concerning processing parameters can be found in [22].

### 2.2. Static Tensile Tests

Tensile specimens of 4.5 mm thickness with a gauge width of 12.5 mm were machined from the thermomechanically processed sheet parallel to the rolling direction to investigate the effect of deformation temperature on the microstructure evolution and stability of retained austenite. The tensile tests were performed at 20–200 °C at a strain rate of 10^−3^ s^−1^. All specimens were strained up to rupture.

### 2.3. SEM and EBSD Analysis

Changes in the microstructure at various deformation temperatures were analyzed using scanning electron microscopy (SEM) and EBSD methods. The initial microstructure (before the tensile test) and specimens deformed up to rupture at various temperatures were observed after 1% nital etching by means of a scanning electron microscope, FEI Inspect F (FEI, Hillsboro, OR, USA), working in a secondary electrons (SE) detection mode. The energy used for the analysis was 15 kV and the working distance was 10 mm. 

In order to determine the amount of retained austenite at rupture conditions, the electron backscatter diffraction (EBSD) method was applied. The investigations were carried out using a high-resolution JEOL JSM 7200F scanning electron microscope (JEOL, Tokyo, Japan). EBSD analyses were performed with: accelerating voltage 15 kV, step size 0.025 µm, investigated area size 5.7 µm ×13 µm, and working distance 15 mm. Samples were prepared by mechanical grinding and then they were electrolytically polished using a TenuPol-5 device (Struers, Ballerup, Denmark) working at a voltage of 17 kV for 30 s. The A3 electrolyte by Struers at temperature 0 °C was used. In this case, it was not necessary to polish the sample until perforation occurred. Hence, the sample polishing for EBSD using this method took a relatively short time (seconds). The EBSD data were post processed using the TSL®OIM (Analysis 8) software.

The main parameters used to identify the retained austenite were the image quality (IQ) and confidence index (CI) factors. The IQ factor represented a quantitative description of the sharpness of the bands in the EBSD pattern. A lattice distorted by crystalline defects, such as dislocations, subgrain boundaries, and internal stresses affected a Kikuchi pattern quality leading to lower IQ values [19,23]. In the present study, the following maps were analyzed: IQ image quality maps, phase distribution maps, crystallographic orientation distribution (inverse pole figure), misorientation angle maps, and kernel misorientation maps.

### 2.4. X-Ray Diffraction

The sample in the initial state was compared to the specimens deformed up to rupture at various temperatures. Measurements of the volume fraction of retained austenite and carbon content in this phase were conducted by means of an Empyrean PANalytical diffractometer (PANalytical, Almelo, The Netherlands) operating in a Theta-Theta arrangement, using simultaneous movement of the lamp and detector, while its structure is based on the Bragg-Brentano principle of diffraction radiation focusing. Measurements were carried out using cobalt radiation with iron filter in configuration with a Pixcel detector. The tube voltage of 40 kV and a tube current of 30 mA were applied. The step size, scanning angle range, and counting time were 0.02626°, 46°–105°, and 300 s, respectively. The comparative Averbach-Cohen method was used to quantitatively determine a volume fraction of retained austenite. This method is commonly used in the case of multiphase steels [24,25]. The lattice parameter of the austenite was determined using the diffraction pattern and next carbon content in γ phase was estimated using Equation (1) taking into account major alloying elements in steel, i.e., Mn and Al [26]:

a_γ_ = 3.556 + 0.0453C_γ_ + 0.00095Mn + 0.0056Al (1)

where a_γ_—lattice parameter of the austenite (Å), C_γ_—carbon content in the austenite (wt.%), Mn, Al—content of alloying elements in steel (wt.%).

## 3. Results

### 3.1. Microstructure in the Initial State

**Figure 1:**
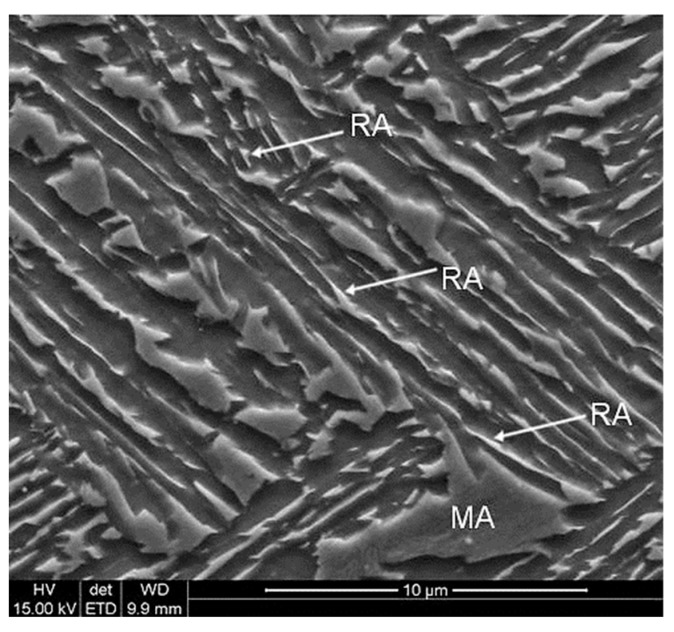
SEM micrograph of the specimen in the initial state (undeformed) characterized by bainitic ferrite laths containing retained austenite (RA) and martensite–austenite (MA) constituents.

Figure 1 shows the microstructure of investigated steel in the initial state. The matrix consisted of fine-lath bainitic ferrite and some fraction of martensite. Since manganese slowed down the austenite-ferrite transformation kinetics strongly no ferrite was formed. Hence, the only austenite was present at 700 °C and it decomposed into bainite during further cooling. Retained austenite occurred in the form of thin layers (RA) located between bainitic laths or as martensitic-austenitic (MA) constituents. MA areas formed as a result of partial martensitic transformation of blocky-type austenite grains due to a lower carbon content [6,16] in comparison to layered austenite (Figure 1). 

**Figure 2:**
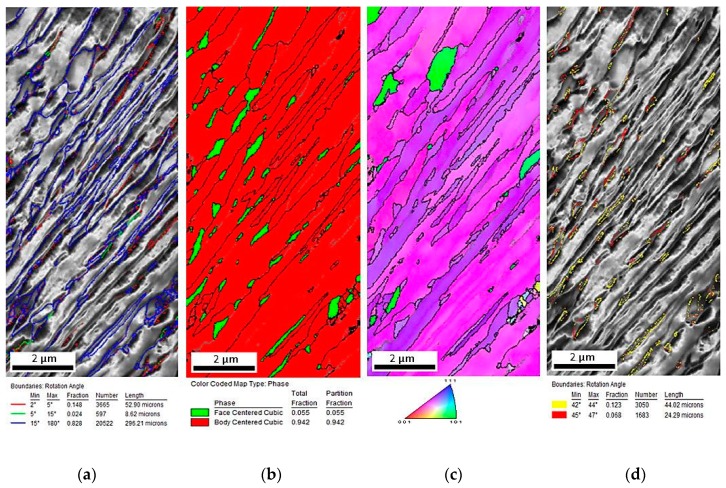
Electron backscatter diffraction (EBSD) analyses of the steel in the initial state. Image quality (IQ) map (**a**), phase distribution map—retained austenite (RA) as green (**b**), orientation map (**c**), map displaying the boundary character between γ and α grains (**d**).

**Figure 3:**
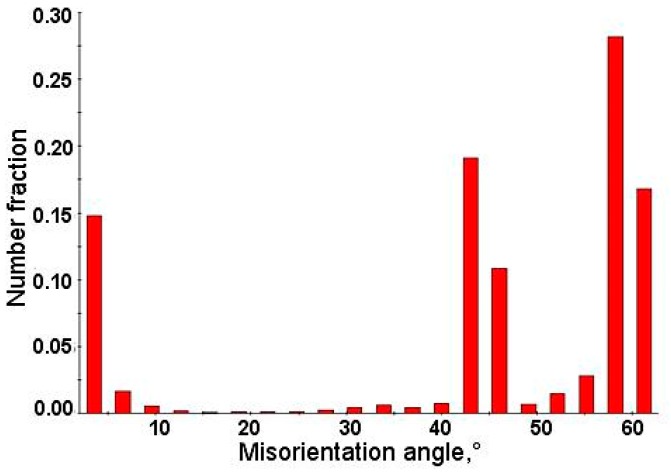
Distribution of misorientation angles in the initial state.

Figure 2a–d provides the EBSD crystallographic analyses of the undeformed steel. Figure 2a shows the image quality (IQ) map. Martensite occurred as the darkest areas characterized by the lowest values of the IQ parameter, whereas areas characterized by a higher intensity were represented by bainitic ferrite. The grains where the martensitic transformation occurred showed a high density of dislocations [27,28]. Therefore, the quality of diffraction in these areas was the lowest. In Figure 2a, retained austenite corresponds approximately with the dark areas. The fraction of low-angle boundaries (2° < misorientation < 15°) marked as red and green was estimated to be 17.2% (Table 1). High-angle boundaries marked as blue (misorientation > 15°) were dominant—82.8%. Figure 2b shows that small austenite grains (the average size about 0.22 µm) can be observed and they were mainly located at high-angle grain boundaries (Figure 2a). It can be observed on the phase distribution map (Figure 2b) that retained austenite (marked in green) occurred mainly in a form of thin layers and small grains located at martensitic areas, which were formed upon cooling. Red represents microstructure constituents, which were characterized by a regular, body-centered crystallographic lattice including bainitic ferrite and martensite. Based on the crystallographic orientation map (Figure 2c) it was found that retained austenite was characterized mainly by the <111> direction of crystallographic orientation. In Figure 2d, the bainitic ferrite/martensite–retained austenite interfaces were highlighted in different colors, which represented the interface deviating from the Kurdjumov–Sachs (yellow) of (111)_γ_//(011)_α_ and [101]_γ_//[111]_α_ and Nishiyama–Wassermann (red) of (111)_γ_//(011)_α_ and [112]_γ_//[011]_α_ orientation relationships [27]. The occurrence of angles close to 45° in a misorientation angle distribution histogram (Figure 3) confirmed the presence of Kurdjumov–Sachs and Nishiyama–Wassermann relationships between bainitic ferrite or martensite and retained austenite. Similar results were also observed by other authors in TRIP-aided steels [27,28]. A fraction of grain boundaries showing the Kurdjumov–Sachs crystallographic relationship with the bainitic ferrite was 12.3% and 6.8% for the Nishiyama–Wassermann relationship.

**Figure 4:**
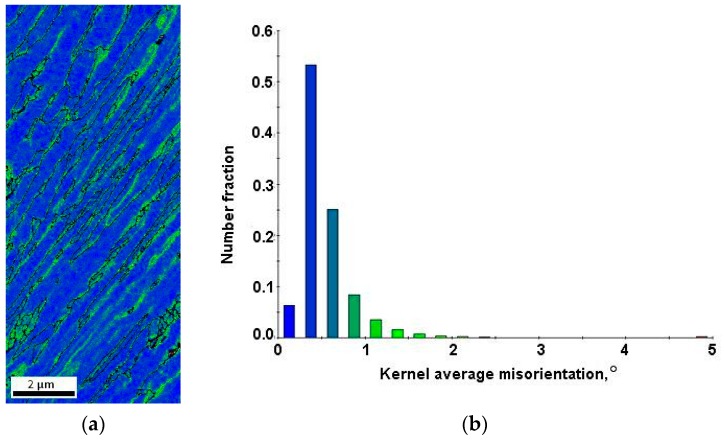
Color-coded kernel average misorientation map displaying the local strain distribution in the specimen in the initial state (**a**) and kernel average misorientation distribution (**b**).

Figure 4a displays the local strain distribution of the non-deformed sample by means of the color-coded kernel average misorientation map. The map was calculated to a maximum misorientation of 5°. Blue shows misorientations less than 1° and green represents pixels that were disoriented with respect to the kernel higher than 1°. The results presented in Figure 4a revealed that the remaining plastic strain was preferentially localized at grain boundaries, especially in fine-grained austenitic–martensitic areas. To quantify the local strain distribution among the phases the average local misorientations were calculated. From the results shown in Figure 4b, it can be seen that in the undeformed sample plastic strains were localized mainly in the retained austenite and martensite areas (average misorientation higher than 1°).

**Figure 5:**
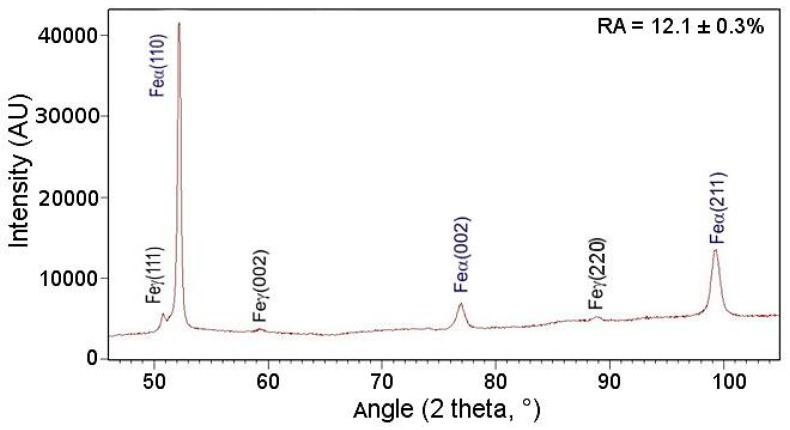
X-ray diffraction pattern of investigated steel in the initial state.

X-ray diffraction analysis was carried out to identify the phases and to determine the retained austenite amount and its carbon content. Figure 5 displays the diffraction pattern of investigated steel in the initial state. The {111}, {002}, and {220} γ phase reflections (face-centered cubic (fcc) structure) and the {110}, {002}, and {211} α phase reflections (body-centered cubic (bcc) structure) were detected. The amount of retained austenite in the initial state was estimated to be 12.1%, while the carbon content in this phase was about 1.1%.

### 3.2. Effect of Deformation Temperature on the Microstructure and Mechanical Stability of Retained Austenite

**Figure 6:**
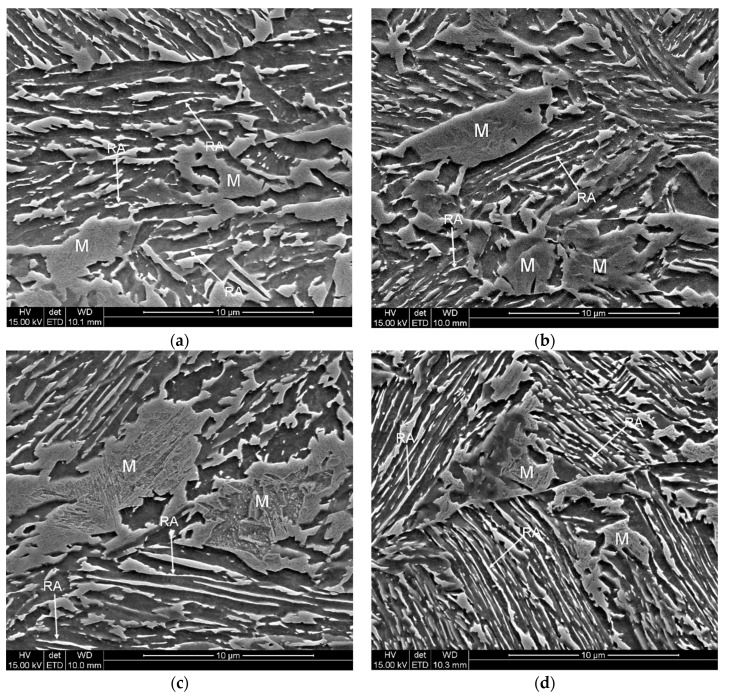
SEM micrographs of the specimens deformed at different temperatures: 20 °C (**a**), 60 °C (**b**), 100 °C (**c**), 200 °C (**d**).

A detailed analysis of the microstructure evolution during deformation conducted at different temperatures allowed the characterization of the mechanical stability of retained austenite. Microstructures of the investigated samples are shown in Figure 6a–d. The microstructure of a steel deformed at 20 °C was characterized by the presence of a small fraction of retained austenite, which can be observed as bright areas (Figure 6a). In this case, RA occurred as thin layers located between bainitic ferrite or a very small part occurred in outer MA areas. Freshly formed strain-induced martensite laths divided the retained austenite grains into smaller blocks, which may contribute to the increase of its mechanical stability. A huge fraction of the γ phase transformed into martensite during straining. Thus, numerous martensitic areas could be observed in the microstructure. It was related to the low stability of γ phase at this deformation temperature. Note that the stability of retained austenite should be discussed by using both amount of remaining retained austenite and plastic strain. The plastic strain values at rupture conditions were similar ~12.2%–14.0% at 20, 100, and 200 °C. Therefore, the results can be directly compared. In steel deformed at 60 °C a slightly higher fraction of RA was observed in the microstructure (Figure 6b). One of the reasons was a smaller value of total elongation (~9.4%) and a corresponding smaller deformation extent, in which the strain-induced transformation could proceed. Similarly to the specimen deformed at 20 °C, only thin layers of retained austenite remained stable. Increasing the deformation temperature to 100 °C resulted in a further increase in stability of RA (Figure 6c). The amount of bright areas represented by the γ phase became higher. The highest amount of retained austenite can be observed in the microstructure deformed at the highest temperature—200 °C (Figure 6d). Numerous thin layers of RA located between bainitic ferrite and austenitic-martensitic areas remained stable. Luo et al. [29] also reported that at low deformation temperatures the volume change of austenite was larger than that at higher temperatures. Some martensitic areas observed in specimens deformed at different temperatures were characterized by the presence of substructure which can be observed as dark areas inside the martensitic-austenitic constituents (Figure 6a–d). 

**Figure 7:**
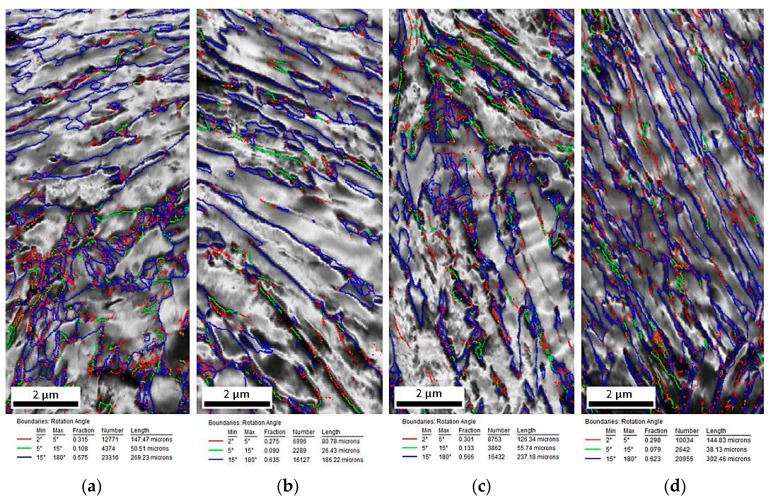
Image quality (IQ) maps of the steel deformed at: 20 °C (**a**), 60 °C (**b**), 100 °C (**c**), and 200 °C (**d**).

The EBSD measurements were performed to characterize the changes in the microstructure of investigated steel at different deformation temperatures. The EBSD method led to the determination of the size, shape, and distribution of retained austenite. Figure 7a–d shows the image quality (IQ) maps, which represent a quantitative description of the sharpness of the electron backscatter diffraction pattern. The fraction of low-angle boundary (2° < misorientation < 15°), marked in red and green (Figure 7a–d), was higher for the deformed specimens when compared to the specimen in the initial state (Figure 2a). These boundaries formed usually inside or around the austenitic–martensitic areas [30]. Their amount rose from 17.2% detected in the initial state, to circa 43.4% for the specimen deformed at 100 °C (Table 1). It indicated indirectly a significant increase in the dislocation density during plastic deformation. High-angle boundaries marked in blue (misorientation > 15°) were in a minority for the specimen deformed in the temperature range 20–200 °C in comparison with the base specimen (Table 1). In general, all structural defects (such as dislocations) caused a crystal lattice distortion [21,27]. Therefore, on the one hand, dislocations generated during plastic deformation were reflected as an increase in the number of low-angle boundaries. On the other hand, the new-formed martensite–austenite boundaries due to strain-induced generated new high-angle boundaries. Since, the total number of low-angle and high-angle boundaries was 100%, the final partition between low-angle and high-angle boundaries was a synergic impact of these two effects.

Moreover, the average grain size of RA was lower for deformed specimens. It was related to the fragmentation of retained austenite grains into smaller blocks by newly formed strain-induced martensite. The smallest average grain size of RA showed the specimen deformed at 20 °C (Table 1), in which the martensitic transformation occurred the most intensively due to the lowest stability of the γ phase.

**Figure 8:**
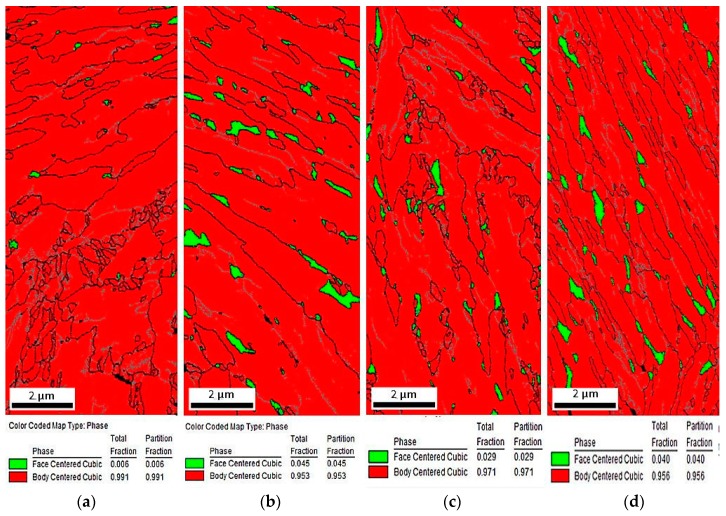
Phase distribution maps (retained austenite (RA) in green) of the investigated steel deformed at: 20 °C (**a**), 60 °C (**b**), 100 °C (**c**), and 200 °C (**d**).

Phase distribution maps displayed in Figure 8a–d show that the amount of retained austenite (marked in green) was related to the deformation temperature. The typical width of layered retained austenite was in the range 0.1–0.2 µm (SEM images). Since, the scanning step is 25 nm they should be detected. In practice, the identification of layered retained austenite in the deformed samples was more difficult compared to non-deformed samples due to worse conditions for Kikuchi pattern’s completion. Therefore, the EBSD results should be treated as an additional confirmation of the general tendency observed using more reliable XRD results. The smallest amount of RA was detected in the specimen deformed at 20 °C, whereas specimens deformed at higher temperatures possessed a higher fraction of RA (Table 2). Only a few very small grains of retained austenite were detected at 20 °C (Figure 8a). The highest fraction of RA was identified in specimens deformed at higher temperatures (Figure 8b–d). Retained austenite occurred in the form of thin layers located between bainitic ferrite and small grains located in martensitic areas resulting from the fragmentation of larger austenite grains.

**Figure 9:**
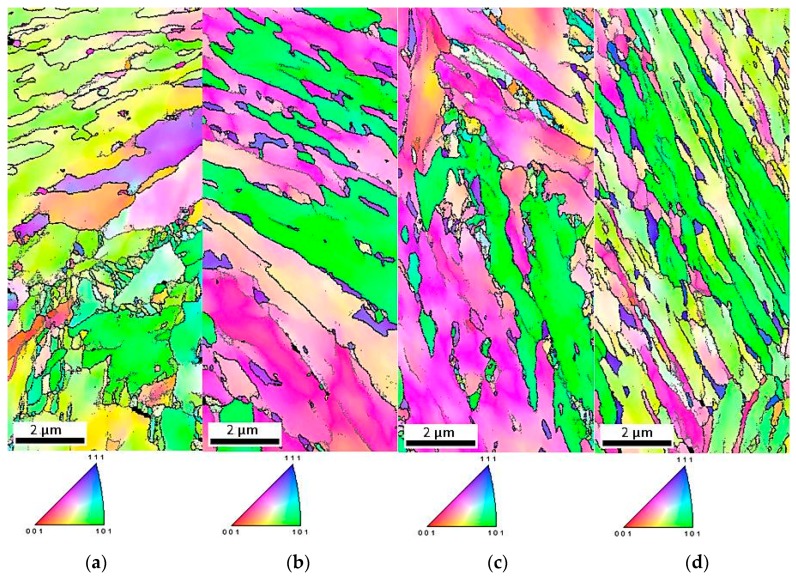
Inverse pole figures of the steel deformed at: 20 °C (**a**), 60 °C (**b**), 100 °C (**c**), and 200 °C (**d**).

**Figure 10:**
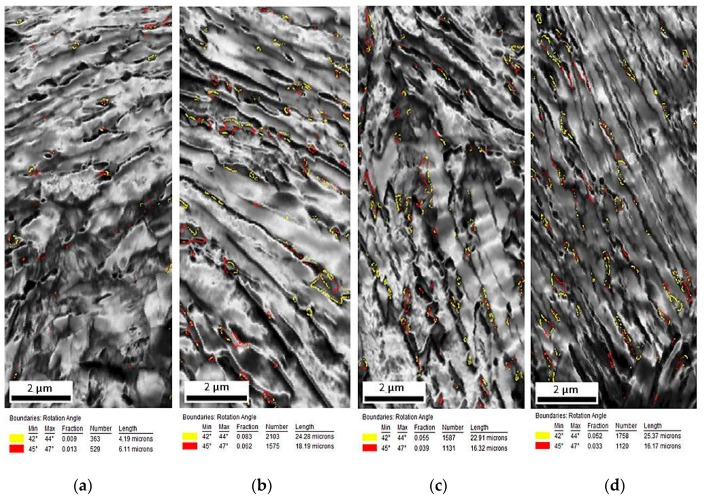
Maps displaying the boundary character between γ and α grains in the investigated steel deformed at: 20 °C (**a**), 60 °C (**b**), 100 °C (**c**), and 200 °C (**d**).

**Figure 11:**
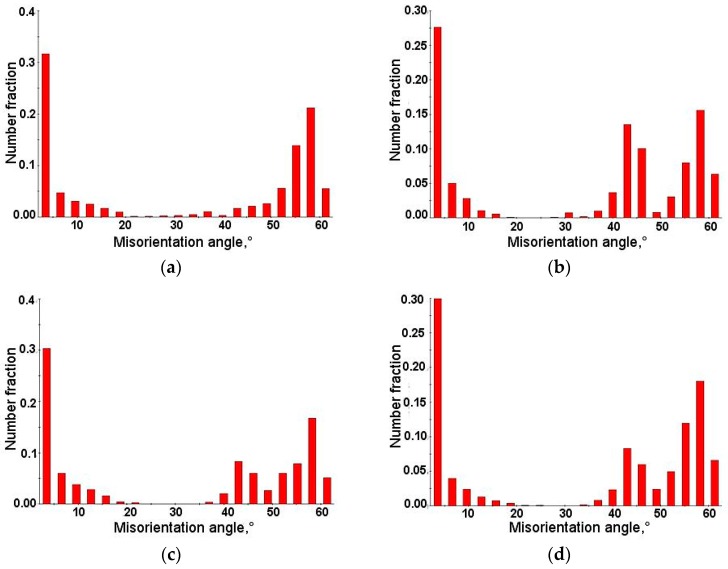
Distribution of misorientation angles in the specimens deformed at: 20 °C (**a**), 60 °C (**b**), 100 °C (**c**), and 200 °C (**d**).

**Figure 12:**
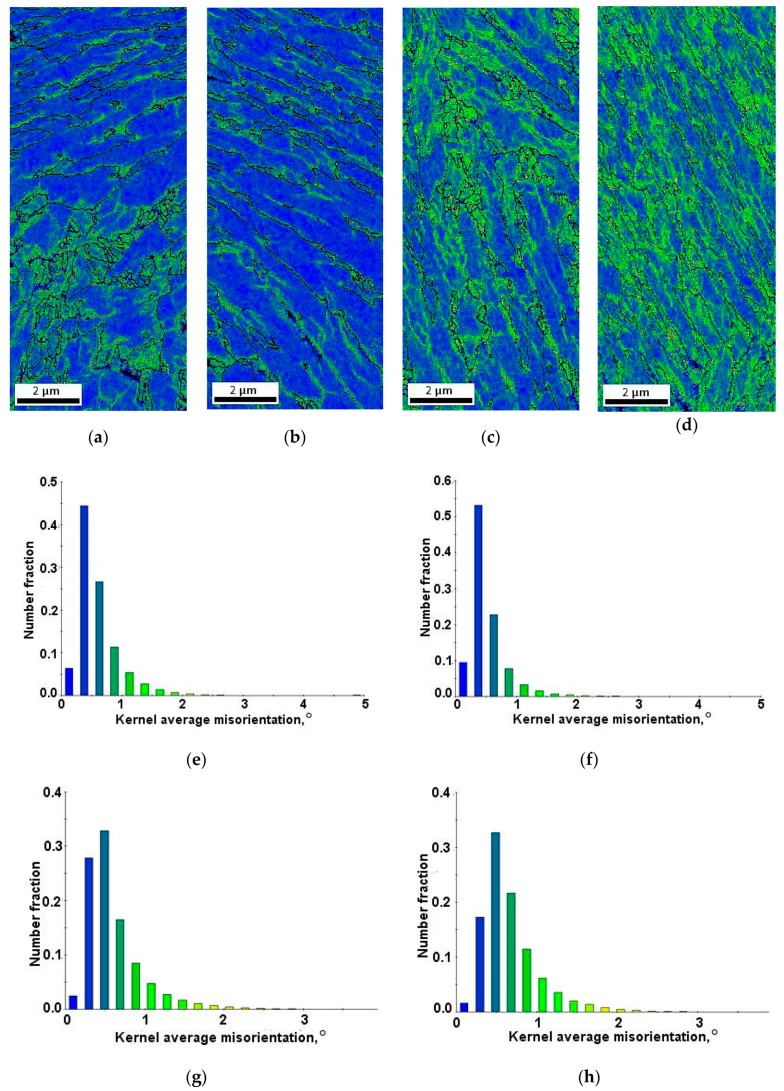
Color-coded kernel average misorientation maps displaying the local strain distribution and kernel average misorientation distribution for the specimens deformed at: 20 °C (**a**,**e**), 60 °C (**b**,**f**), 100 °C (**c**,**g**), 200 °C (**d**,**h**).

Inverse pole figures in Figure 9a–d show that retained austenite was still characterized by the <111> direction of crystallographic orientation. Moreover, deformed specimens showed greater differentiation of crystallographic orientation than the undeformed specimen (Figure 2c). The amount of retained austenite in deformed specimens was lower than that in the initial state. Thus, the fraction of γ phase showing crystallographic relationships with the bainitic ferrite and newly formed martensite decreased (Figure 10a–d). The fraction of grain boundaries showing the Kurdjumov-Sachs and Nishiyama-Wassermann relationships was the lowest in the specimen deformed at 20 °C (Figure 11a–d). Figure 12a–d displays the local strain distribution in the deformed samples by means of the color-coded kernel average misorientation (KAM) maps. Obtained results showed that the quantity of areas showing a kernel misorientation above 1° was higher than that for the non-deformed specimen (Figure 4a,b). It was related to the microstructure distortion during straining. KAM was sensitive to the dislocation characteristics and their density [5]. The highest strain localization showed the fine-grained austenite in combination with newly formed martensitic areas (Figure 12a–d). It was due to the deformation-induced martensitic transformation of the austenite. However, in the specimens deformed at 100 and 200 °C the amount of kernel misorientation higher than 1° was higher than at lower deformation temperatures (Figure 12c,d). It can be related to the higher amount of RA areas. Dutta et al. [31] reported that retained austenite islands showed higher stress localization than martensite. They also noted that strain localization led to faster strain-induced martensitic transformation of the γ phase to α’. The strain partitioning between austenite and martensite was reported to be responsible for the enhanced plasticity of medium-Mn steels.

**Figure 13:**
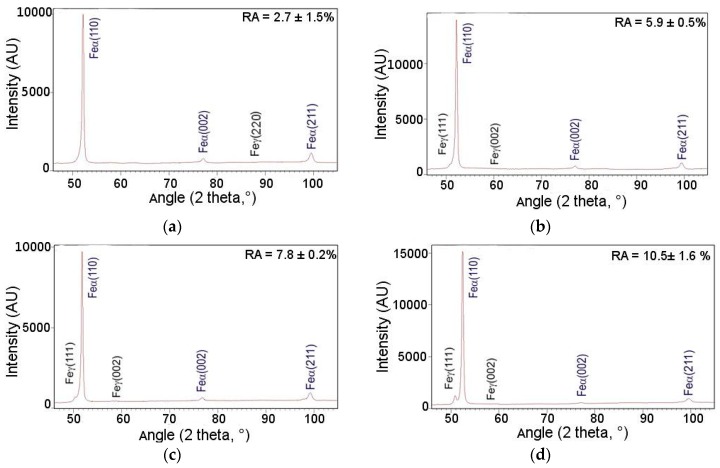
X-ray diffraction patterns of investigated steel deformed at: 20 °C (**a**), 60 °C (**b**), 100 °C (**c**), 200 °C (**d**).

XRD measurements revealed the presence of diffraction lines from α and γ phases. Detailed information on the fraction of retained austenite obtained using the XRD and EBSD methods and corresponding carbon contents in this phase are listed in Table 2. The obtained results are also presented in Figure 13a–d. The changes in the amount of retained austenite depending on the deformation temperature were discussed according to the values of total elongation presented in Table 2. The initial volume fraction of RA in the investigated steel was 12.1%. Results of XRD analysis showed that a volume fraction of retained austenite decreased with increasing deformation temperature. It should be noted that the retained austenite stability was also related to the extent of strain. According to the results in Table 2, the plastic strain for almost all temperatures was similar. Only at 60 °C, the total elongation was a little bit smaller. It can be due to a higher amount of retained austenite measured by the EBSD method at this temperature. The lowest amount of retained austenite was at 20 °C (2.7%), whereas the highest fraction was detected in the specimen deformed at 200 °C (10.5%). It was related to a gradual increase in the stability of RA with increasing deformation temperature. Results obtained by XRD were in relatively good agreement with microstructural observations (Figure 6a–d and Figure 8a–d). EBSD results showed typical discrepancies. However, the XRD tendency was confirmed.

In addition to the temperature effect, the stability of retained austenite was also affected by chemical composition, especially carbon content in this phase and its morphology [4,14,15,16,17,18]. The bainitic transformation at 400 °C for 300 s provided only the interstitial diffusion of C since Mn required a vacancy diffusion. Therefore, the C content in the austenite was critical for the mechanical stability of retained austenite, whereas Mn was considered as a bulk content. The highest carbon content showed retained austenite observed in the specimen deformed at 20 °C (1.15%). Only the smallest grains located at martensitic areas and thin layers of γ phase stayed stable after deformation (Figure 6a–d and Figure 8a–d). It was related to the fact that thin layers of retained austenite were characterized by a higher carbon content than blocky-type austenite [6,16]. In the case of higher deformation temperatures, the carbon content in retained austenite was similar, i.e., 1.05%–1.08%. This was due to the higher amount of blocky retained austenite, which was characterized by a lower average carbon content than film-type austenite [29].

## 4. Discussion

**Figure 14:**
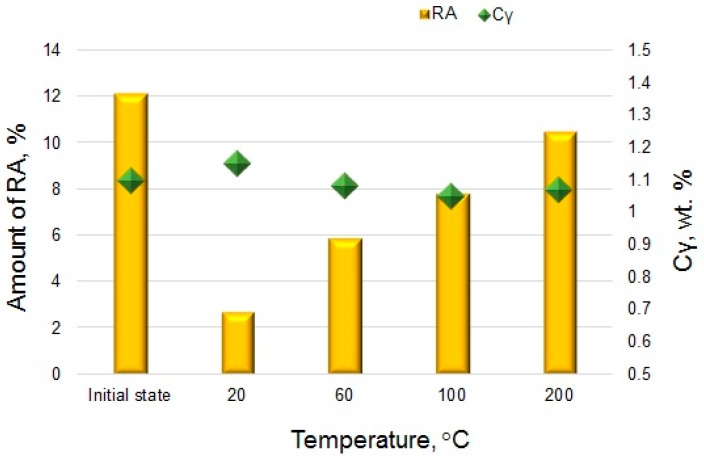
Fraction of retained austenite and its carbon content obtained by XRD analysis for steel in the initial state and deformed at 20, 60, 100, and 200 °C.

The effect of deformation temperature on the microstructure evolution and stability of retained austenite has barely been discussed in literature, especially in case of hot-rolled medium manganese steels. Investigations concerning the stability of RA in such steels were focused primarily on the influence of room-temperature deformation on microstructure–property relationships [1,3,8]. A detailed characterization of the stability of RA at elevated temperatures is important due to its influence on the TRIP effect and corresponding mechanical properties of final steel products. So far, similar experiments were performed by other authors in multiphase steels composed of ferrite, bainite, and retained austenite [17,32]; high-Mn steels showing a TRIP effect [33]; quenching and partitioning steels [34]; and cold-rolled medium-Mn steels with a ferritic–austenitic microstructure [18]. Independently, on a TRIP steel grade, a similar tendency was observed. The intensity of the TRIP effect decreased with an increase in deformation temperature. It was related to the increase of the stacking fault energy (SFE) value resulting in a higher stability of retained austenite [4]. Results obtained in the present study were in good correlation with data presented in literature [17,32,33,34]. The smallest amount of RA was detected by XRD and EBSD methods in the specimen deformed at 20 °C (2.7% and 0.6%, respectively), whereas the highest fraction of retained austenite was identified at 200 °C (10.5%)—Figure 14. It can be concluded that an M_d_ temperature above which martensitic transformation was not possible to occur was not achieved. This meant that the M_d_ was a little bit higher than 200 °C because only circa 1.6 vol.% of RA transformed into martensite at this temperature (Table 2).

The amounts of RA obtained by the XRD method were somewhat different when compared to the results of the EBSD measurements (Table 2). This was typical [21,28] because the EBSD method was able to give more detailed and accurate information on retained austenite amount and morphology on a local scale but the results collected at high magnifications cannot be considered as reliable due to poor data statistics and local observations [21]. EBSD analysis usually detected the information at the surface of specimen. However, in XRD analysis, the penetration depth of X-ray was around 10 μm. It implied that volume fraction of retained austenite was measured at circa 10 μm inside from the surface in XRD analysis. For retained austenite located at the surface of specimen (EBSD technique), there was no restraint force because retained austenite was not surrounded by the bainitic ferrite matrix. This fact meant that retained austenite was easily transformed to martensite. Therefore, the volume fraction of retained austenite measured by EBSD was less than that measured by XRD. From this point of view, the information collected by XRD measurements provided better quantitative information on retained austenite amounts over an entire examined volume.

The stability of retained austenite depended substantially on its chemical composition (especially carbon content), morphology, and the plastic strain value applied. Taking into account that the total elongation values for almost all temperatures were similar (except 60 °C), the results can be successively compared. Thin layers of RA located between bainitic ferrite were more stable than blocky-type grains due to a higher carbon content [34,35]. Carbon strongly affected the austenite stabilization and decreased the M_s_ temperature. Pereloma et al. [4] reported that the most favorable, a gradual progress of the strain-induced transformation occurred when retained austenite contained 1.1%–1.6% C. In the present study, the carbon content in the γ phase was in the range 1.05%–1.15%. This meant that 1.1 wt.% was an average value of all grains population for the initial state, whereas other C contents determined averaged values for a remaining part of untransformed γ grains. Therefore, the highest carbon content (1.15 wt.%) was detected at 20 °C (Table 2). In this case, a huge part of retained austenite transformed into martensite. The remaining austenite occurred only as thin layers between bainitic laths and new-formed strain-induced martensite (Figure 6a). Stabilization of the γ phase was additionally supported by hydrostatic pressure introduced by the bainitic ferrite and freshly formed martensitic laths, which divided retained austenite grains into smaller blocks [35,36]. That was why the C content in retained austenite at 20 °C was the highest (Figure 14). In the deformation range 60°–200° the carbon content was a little bit lower compared to the initial state due to a higher population of blocky retained austenite grains remaining untransformed in the microstructure.

The monitoring of strain-induced transformation at different temperatures using EBSD revealed that the deformed microstructures were characterized by a higher population of low-angle grain boundaries (Figure 7) compared to the undeformed specimen. It was due to the substructure development and a synergic competition between new-generated high-angle martensite–austenite boundaries and numerous new-generated dislocations associated with low-angle boundaries [21,27]. The strain-induced martensite formation introduced some new high-angle grain boundaries (Figure 8). However, their relative amount was lower compared to new-formed low-angle boundaries being a consequence of straining. The kernel results (Figure 12) indicated that the strain gradient increased with increasing deformation temperature. Typically, this was the most advanced at grain boundaries and within strain-induced martensite due to highest stresses at these areas [7,21]. It was interesting that the strain gradient was most developed at 200 °C showing that strain was rather distributed in the retained austenite and neighboring bainitic ferrite laths instead of recovery-stimulated strain decrease, which could take place at elevated temperatures. Thus, the kinetics of strain-induced martensitic transformation at elevated temperatures was a complex issue as was the interplay between different structural constituents showing different stress levels during straining.

## 5. Conclusions

The work addressed a detailed study of the microstructure evolution and stability of retained austenite in TRIP-assisted 0.17C-3.1Mn-1.6Al-0.22Si-0.22Mo-0.04Nb steel deformed up to rupture in static tensile tests performed at the temperature range 20–200 °C. The microstructural analysis was done by means of SEM observations, EBSD, and XRD methods to obtain a better understanding of the strain-induced martensitic transformation and thus changes in a volume fraction of retained austenite at elevated temperatures. The main findings of the present study were as follows:

- Temperature of the plastic deformation significantly affected the stability of retained austenite. An increase in the deformation temperature resulted in reduced development of the TRIP effect due to its higher mechanical stability. The highest fraction of RA transformed into martensite took place at 20 °C, whereas the lowest intensity of martensitic transformation was observed at 200 °C.
- Stability of retained austenite was primarily influenced by its morphology and carbon content. RA in the form of thin layers located between bainitic ferrite laths was characterized by higher stability than blocky retained austenite due to the higher carbon content.
- Stabilization of the γ phase was supported by bainitic ferrite and the hydrostatic pressure introduced by the freshly formed martensitic laths which divided retained austenite grains into smaller blocks. The smallest average grain size of RA showed the specimen deformed at 20 °C, which experienced the most intense martensitic transformation.
- The fraction with a low-angle boundary was higher for the deformed specimens compared to the initial state. These boundaries formed usually within or near martensitic–austenitic constituents.
- The untransformed austenite and neighboring parts of bainitic ferrite were deformed more intensively at elevated temperatures (100–200 °C) compared to central parts of bainitic ferrite, which was confirmed by kernel misorientation analyses.

## Figures and Tables

**Table 1:** Morphological parameters of retained austenite at different deformation temperatures determined using the EBSD method.

Morphological Parameters	Initial State	Deformation Temperatures			
		20 °C	60 °C	100 °C	200 °C
**Average Grain Size of RA**	0.22 µm	0.10 µm	0.18 µm	0.16 µm	0.21 µm
**Low-Angle Boundaries**	17.2%	42.3%	36.5%	43.4%	37.7%
**High-Angle Boundaries**	82.8%	57.5%	63.5%	56.6%	62.3%

**Table 2:** Average amount of retained austenite at different deformation temperatures determined using EBSD and XRD methods, carbon content in RA, and corresponding total elongation values.

Deformation Temperatures, °C	Fraction of Retained Austenite, vol.% (XRD)	Fraction of Retained Austenite, % (EBSD)	Carbon Content in Retained Austenite, wt.%	Values of Total Elongation, %
20	2.7 ± 1.5	0.6	1.15 ± 0.08	14.0 ± 1.3
60	5.9 ± 0.5	4.5	1.08 ± 0.03	9.4 ± 1.0
100	7.8 ± 0.2	2.9	1.05 ± 0.05	12.3 ± 1.1
200	10.5 ± 1.6	4.0	1.07 ± 0.02	12.2 ± 1.4
Initial state	12.1 ± 0.3	7.6	1.10 ± 0.03	-
